# Clinical Utility of lncRNAs in Urothelial Carcinoma of the Bladder: Diagnostics, Risk Stratification, and Treatment Guidance

**DOI:** 10.3390/ijms27219461

**Published:** 2026-10-23

**Authors:** Janusz Ławiński, Beata Franczyk, Abdusalam Sagr, Karolina Popek, Jacek Tabarkiewicz

**Affiliations:** 1 Faculty of Medicine, University of Rzeszów, Rejtana 16C, 35-959 Rzeszow, Poland; 2 Department of Nephrocardiology, Medical University of Lodz, Zeromskiego 113, 90-549 Lodz, Poland; 3 Nikolay Pirogov Specialized Hospital in Lodz, Wolczanska, 90-531 Lodz, Poland; 4 Laboratory for Translational Research in Medicine, Centre for Innovative Research in Medical and Natural Sciences, University of Rzeszów, 35-959 Rzeszow, Polandj.tabarkiewicz@ur.edu.pl (J.T.); 5 Department of Human Immunology, Faculty of Medicine, College of Medical Sciences, University of Rzeszów, 35-959 Rzeszow, Poland

**Keywords:** bladder cancer, urothelial carcinoma, long non-coding RNA (lncRNA), urinary biomarkers, liquid biopsy, urine cytology, urinary extracellular vesicles, exosomes, non-muscle-invasive bladder cancer (NMIBC), muscle-invasive bladder cancer (MIBC), hematuria, recurrence, progression, risk stratification, surveillance

## Abstract

Urothelial carcinoma of the bladder remains challenging to assess in real time. Standard tools such as white-light cystoscopy and urine cytology are essential, but both have recognized limitations, particularly for carcinoma in situ, low-grade disease and repeated long-term surveillance in non-muscle-invasive bladder cancer (NMIBC). Conventional risk models also show imperfect performance in contemporary practice, contributing to both overtreatment and delayed escalation in biologically aggressive disease. Long non-coding RNAs (lncRNAs) are emerging as attractive biomarkers because of their context-specific expression, measurable expression in tumor tissue and detectability in urine and blood, including within extracellular vesicles (EVs), such as exosomes that protect RNA from degradation. The potential clinical role of lncRNAs is determined by the specimen, molecular compartment, disease setting, and intended use. Urinary cell-free and urinary EV-associated lncRNAs currently have the strongest translational evidence for hematuria triage diagnostic support, recurrence-risk assessment, and longitudinal molecular monitoring. Tissue lncRNAs are most relevant to biological classification and prognostic refinement after transurethral resection or cystectomy, whereas blood-based lncRNAs may provide complementary information on systemic disease burden and treatment-associated response, particularly in muscle-invasive or advanced disease. Although several urinary multi-lncRNA panels have functioned as stand-alone statistical classifiers in development cohorts, no lncRNA assay has been prospectively validated to replace cystoscopy, cytology, histopathology, or established treatment-selection biomarkers in routine care. Repeatedly studied candidates include UCA1 and several exosomal/uEV-associated lncRNAs (e.g., *TERC, SNHG16*), while multi-lncRNA panels and combined strategies (including integration with established urine tests such as NMP22) may stabilize performance across biological and pre-analytical variability. Evidence also supports the concept of longitudinal “molecular response” monitoring using liquid-based lncRNA readouts, although truly predictive, treatment-interaction evidence remains limited. Across studies, performance is strongly influenced by the urine compartment analyzed (pellet/sediment, cell-free urine, or urinary EVs (uEVs)/exosomes) and by pre-analytical handling and normalization choices. lncRNA assays are unlikely to replace cystoscopy in the near term, but they have clear potential as decision-support tests that complement clinical risk assessment, cytology, and other urine-based platforms. This work synthesizes mechanistic and clinically annotated evidence on lncRNAs in bladder cancer, with an emphasis on urine-based and uEV/exosomal assays. The search strategy covered publications from 1 January 2015 to 30 December 2025, with emphasis on most recent clinically relevant studies, and included mechanistic studies with functional validation, liquid biopsy/urine studies, and evidence syntheses.

## 1. Introduction

Bladder cancer (BC) is the ninth most commonly diagnosed malignancy and continues to pose a great health and economic burden because of its high incidence, male predominance, and chronic relapsing natural history. According to recent global estimates for 2022, there were >600,000 new cases and >200,000 deaths worldwide, with substantial geographic variation [1]. Tobacco smoking remains the dominant modifiable risk factor in most regions. Occupational exposure to aromatic amines and contamination of drinking water with arsenic are additional established risks, while infection-related pathways (e.g., schistosomiasis) contribute to distinct epidemiological and histological patterns in some endemic settings (sub-Saharan Africa) [1,2]. Clinically, approximately three-quarters of patients present with non-muscle-invasive bladder cancer (NMIBC), whereas the remainder present with muscle-invasive bladder cancer (MIBC) or metastatic disease [3]. These subtypes follow divergent clinical pathways with markedly different treatment intensity and prognosis [4]. Reliable real-time assessment of this disease remains challenging: diagnostic sensitivity varies across modalities, longitudinal monitoring is burdensome, and the risk of progression is often recognized only after clinically meaningful time has been lost, which complicates timely treatment escalation [5,6,7]. Recent analyses confirm that even updated risk groupings may fail to accurately predict high-grade recurrence-free and progression-free outcomes in bacillus Calmette-Guérin (BCG)-treated populations, emphasizing a persistent gap between trial-derived models and real-world heterogeneity [8,9]. These limitations may contribute directly to the problems of overtreatment and undertreatment. Some patients with indolent, low-grade NMIBC may undergo repeated resections and intensive cystoscopic schedules with marginal clinical benefit, whereas others with biologically aggressive disease may be falsely reassured, delaying escalation (including timely cystectomy in selected BCG-refractory settings) [10,11]. Alongside imperfect risk prediction, the performance constraints of standard surveillance tests remain clinically important. For example, enhanced surveillance can improve lesion detection but may also increase false-positive findings, reinforcing the need for better risk-adapted strategies rather than “more testing for all” [12].

In this context, long noncoding RNAs (lncRNAs) are emerging as clinically attractive biomarkers because they can show tissue- and state-specific expression patterns, are measurable with widely available nucleic acid methods, and can be detected in urine, often within extracellular vesicles (EV)/exosomes that protect RNA from degradation [7,13,14]. A growing body of evidence supports urinary lncRNAs as diagnostic and potentially prognostic candidates, and EV-enriched preparations appear particularly relevant for non-invasive surveillance and early detection [13,15]. For example, urinary EV (uEV)-associated *SNHG16* showed greater diagnostic discrimination than urine cytology in a small case–control cohort [16]. Such results justify further development but do not establish superiority in consecutive hematuria or surveillance populations. lncRNA assays should therefore be evaluated as components of integrated diagnostic pathways rather than presumed replacements for established tests. Urinary tumor DNA and targeted sequencing panels are progressing rapidly and provide mutation-level information but remain relatively costly and analytically complex for frequent longitudinal testing [5]. Targeted lncRNA-based tests, especially robust multiplex assays based on cell-free urine or uEVs, may offer a complementary balance between clinical performance, cost and workflow feasibility. Their most plausible roles include triage before cystoscopy, reflex testing after equivocal cytology, and serial risk assessment between scheduled surveillance procedures, provided that sensitivity for high-grade disease and carcinoma in situ is maintained [7,17,18].

The aim of this narrative review is to critically evaluate current biological and clinical evidence for lncRNAs in bladder cancer, with particular focus on specimen-specific interpretation, urine-based and EV-associated lncRNA assays, diagnostic and prognostic performance, treatment-associated evidence, and the requirements for clinical implementation.

## 2. Methods

This structured narrative review was designed to critically evaluate clinically relevant and mechanistically supported evidence on lncRNAs in bladder cancer, with a particular focus on urine-based and urinary EV/exosomal biomarkers and their potential clinical applications. PubMed/MEDLINE was the primary bibliographic database. The principal search covered publications dated from 1 January 2015 to 30 December 2025 and was supplemented by backward reference screening of eligible primary studies and major evidence syntheses. Earlier seminal studies were retained when they were necessary to establish biological mechanisms, assay development, or the historical basis of a clinically relevant biomarker. The core PubMed search combined the following concepts: (“Urinary Bladder Neoplasms”[MeSH] OR “bladder cancer”[Title/Abstract] OR “urothelial carcinoma”[Title/Abstract]) AND (“RNA, Long Noncoding”[MeSH] OR lncRNA*[Title/Abstract] OR “long non-coding RNA”[Title/Abstract] OR “long noncoding RNA”[Title/Abstract]). Targeted searches added terms for urine, urinary sediment, cell-free urine, extracellular vesicle, exosome, serum, plasma, tumor tissue, diagnosis, hematuria, recurrence, progression, prognosis, bacillus Calmette-Guérin, cisplatin, chemotherapy, radiotherapy, immunotherapy, and treatment response. Only English-language reports were included in the detailed clinical synthesis. Eligible evidence included original human studies of diagnostic, prognostic, surveillance, or treatment-associated lncRNA biomarkers; studies using urine, urinary EVs, blood, circulating EVs, or tumor tissue; and mechanistic studies with functional validation directly relevant to urothelial carcinogenesis or treatment resistance. Systematic reviews and meta-analyses were used to contextualize consistency, heterogeneity, and diagnostic performance but were not treated as substitutes for primary-study appraisal. Reports were excluded from detailed synthesis when they concerned another malignancy, did not provide lncRNA-specific findings, lacked an interpretable biological or clinical endpoint, were available only as an abstract with insufficient methods, or duplicated an already represented cohort without adding a distinct endpoint. When publications overlapped, the most complete report was prioritized, and complementary analyses were retained only when they addressed a separate clinical question.

Evidence was synthesized qualitatively according to specimen, intended use, and level of clinical validation. A diagnostic biomarker was defined as a marker used to distinguish the presence or absence of disease. A prognostic biomarker was defined as a marker associated with the probability of an outcome irrespective of a specific treatment. A predictive biomarker was defined as a marker identifying differential benefit or harm from a particular intervention.

## 3. Current Standard of Care in Bladder Cancer and Unmet Needs

Hematuria evaluation underpins the diagnostic pathway for BC, as hematuria remains the most common presenting symptom for urothelial malignancy. At the same time, the prevalence of cancer among patients referred with microhematuria is low, meaning that most patients undergo invasive or resource-intensive tests to exclude disease rather than to confirm it [19]. Contemporary guidance therefore emphasizes a structured, risk-stratified diagnostic workflow that balances the need to avoid missed clinically significant disease (particularly high-grade tumors and carcinoma in situ) against the harms and low yield of indiscriminate cystoscopy [19,20]. The 2025 update of the American Urological Association (AUA)/Society of Urodynamics, Female Pelvic Medicine & Urogenital Reconstruction (SUFU) microhematuria guideline formalizes this approach by incorporating patient and hematuria features into risk categories and outlining where urine-based biomarkers might be considered as adjuncts in selected scenarios rather than as replacements for cystoscopy [20]. In practice, evaluation typically begins with confirmation and contextualization (repeat urinalysis where appropriate, consideration of infection, stones or contamination, and a focused exposure history) followed by risk-aligned investigation. For higher-risk presentations, flexible cystoscopy remains the reference test for bladder evaluation, while upper urinary tract assessment is commonly achieved with imaging that escalates with risk, often ranging from renal-bladder ultrasound to computed tomography (CT) urography when robust exclusion of upper tract malignancy is required [20]. Importantly, health-economic work supports selective intensification in higher-risk strata rather than uniform investigation for all, because risk-aligned pathways can improve cost-effectiveness while limiting unnecessary procedures [21,22]. These realities define a key unmet need at the diagnostic front door—a reliable, non-invasive method to rule out clinically important cancer with very high negative predictive value, while also supporting rule-in prioritization when capacity constraints make rapid triage clinically valuable [20,23,24].

Within both hematuria work-up and post-treatment follow-up, current standard tests have well-described constraints. White-light cystoscopy remains the gold standard for diagnosis and surveillance; however, it is invasive, operator-dependent, and less reliable for subtle or flat lesions such as carcinoma in situ [25,26,27]. It also drives substantial patient burden and system workload when applied repeatedly across a large NMIBC population [27,28]. Urine cytology continues to be used because of its high specificity, particularly for high-grade disease, but it has consistently low sensitivity for low-grade tumors and suffers from interpretive variability [18,29]. Standardization through The Paris System (including recent updates) has improved reporting consistency and risk identification, but borderline categories still generate uncertainty and do not fully overcome biological limitations such as low tumor shedding in low-grade disease [29,30]. Recent studies suggest that artificial intelligence can support cytology interpretation and may improve sensitivity or reporting confidence, but robust external validation and careful workflow integration are essential before these approaches can be adopted widely [31,32,33,34]. As a consequence, many centers still depend on repeated invasive assessment to compensate for imperfect non-invasive tools, reinforcing patient anxiety, disruption to daily life, and cumulative discomfort during surveillance [27,28].

After diagnosis, management pathways diverge by stage. NMIBC is typically treated with transurethral resection followed by risk-adapted intravesical therapy and structured endoscopic surveillance, whereas MIBC often requires multimodal management with radical cystectomy and/or trimodality bladder-preserving strategies [25,35]. In NMIBC, repeated cystoscopies, intravesical instillations, and recurrent endoscopic resections drive cumulative morbidity and sustained resource utilization, contributing to NMIBC being among the most expensive malignancies to manage on a per-patient, lifetime basis [36]. Guideline-recommended surveillance intensity is central to this burden. Risk-stratified schedules recommend concentration of cystoscopy and cytology in the first years after diagnosis, with de-escalation for selected low-risk disease but prolonged (often lifelong) follow-up for higher-risk phenotypes [25]. Even when oncologically justified, intensive surveillance has meaningful downstream consequences for patients, including pain and discomfort, procedural anxiety, embarrassment, disruption to work, and the cumulative “scanxiety” of repeated testing and waiting for results, while also driving substantial health-system costs [27,28]. Economic analyses demonstrate that surveillance and treatment costs rise sharply with risk category, and modeling studies suggest that carefully selected de-escalation strategies in high-risk NMIBC could reduce costs with minimal impact on survival outcomes, underscoring the need for more precise tools to safely individualize follow-up [36,37,38].

A further unmet need is risk prediction that performs reliably in modern practice. Tools used to guide treatment and surveillance including the European Organisation for Research and Treatment of Cancer (EORTC) and Club Urológico Español de Tratamiento Oncológico (CUETO) risk models and the European Association of Urology (EAU) risk stratification system have imperfect translation into contemporary cohorts, with validations reporting limited discrimination and systematic miscalibration, often overestimating recurrence and/or progression risks in populations treated according to current standards [39,40,41]. This uncertainty contributes directly to both overtreatment and undertreatment: some patients with indolent disease undergo repeated invasive procedures with limited clinical benefit, while others with biologically aggressive tumors may be falsely reassured, delaying timely escalation. In this broader context, the most realistic near-term goal for urine-based assays is not replacement of cystoscopy, but adjunctive deployment in clearly defined roles. These include hematuria triage to reduce low-yield cystoscopy, prioritization of patients most likely to harbor clinically significant disease, and reflex testing after equivocal cytology, use cases that align with guideline emphasis on structured, risk-adapted care and that can be evaluated in intended-use populations rather than idealized case–control settings [20,23,42]. Multimodal approaches may be particularly attractive, as lncRNA signals measured in urine or urinary extracellular vesicles could complement existing markers and clinical risk factors, provided thresholds are transparent and performance is reported with grade- and carcinoma in situ (CIS)-specific details in real hematuria and surveillance cohorts [24,43,44,45]. There is increasing biological interest in CIS as a molecularly distinct precursor state, and multi-omics profiling studies highlight potentially actionable pathways and signatures in CIS that could, in time, inform urinary detection strategies as well as therapy selection [46].

## 4. Long Non-Coding RNAs as Clinical Biomarkers

Long non-coding RNAs are RNA transcripts typically >200 nucleotides that do not encode proteins but can regulate gene expression through diverse mechanisms, including chromatin modulation, transcriptional control and post-transcriptional regulation. Their biological features make them particularly relevant to urothelial carcinogenesis, because the same lncRNA can influence several cancer hallmarks depending on its subcellular localization, structure, and binding partners. These properties help explain why lncRNA dysregulation can be tightly linked to tumor phenotype, recurrence risk, and treatment response, while still being measurable at the RNA level in clinical material [47]. Nuclear lncRNAs commonly participate in chromatin regulation by recruiting or scaffolding chromatin-modifying complexes at specific genomic loci, thereby shaping histone marks and transcriptional accessibility [48,49,50]. In NMIBC, the lncRNA *UNMIBC* (up-regulated in NMIBC) was shown to physically associate with polycomb repressive complex 2 (PRC2) components (including Enhancer of Zeste 2 [EZH2] and Suppressor of Zeste 12 [SUZ12]) and alter H3K27 methylation at target genes, linking a clinically measurable transcript to a plausible epigenetic mechanism [51]. Clinically, this matters because chromatin-linked lncRNA effects tend to align with durable phenotypes such as proliferation capacity, lineage programs, and long-term recurrence risk or progression trajectories [52]. Beyond chromatin, lncRNAs can exert transcriptional control in two complementary directions. First, they can regulate transcription of downstream genes (often by guiding chromatin regulators or interacting with transcription factors and co-regulators) [52,53]. Second, their own expression is frequently driven by oncogenic transcription factors and microenvironmental cues, which makes them informative readouts of pathway activity [52,54,55,56]. In the cytoplasm (and within extracellular vesicles), many bladder cancer-associated lncRNAs operate through miRNA sponging (competitive endogenous RNA behavior), sequestering miRNAs and de-repressing miRNA targets [57]. This can amplify oncogenic signaling through coordinated derepression of multiple mRNA targets that sit downstream of the same miRNA. LncRNAs can also function as RNA-protein scaffolds, organizing ribonucleoprotein complexes that influence transcription, RNA stability, or signaling [58]. Importantly, these “mechanism classes” overlap in practice: localization can be dynamic, isoforms can differ in binding capacity, and lncRNAs can couple epigenetic and post-transcriptional regulation. This mechanistic flexibility helps explain why lncRNAs can mirror clinically meaningful tumor states and transitions, including early progression and recurrence, while remaining measurable with nucleic-acid assays.

A key reason lncRNAs are attractive biomarkers is their tissue- and context-specific expression, which can provide higher biological specificity than many protein-coding transcripts. However, their clinical interpretation depends on the sampling matrix and the molecular compartment in which the RNA is measured. In biofluids such as urine and blood, extracellular RNA is exposed to abundant RNases, yet lncRNA signals persist through encapsulation within EVs, including exosomes, or associations with ribonucleoprotein or other non-vesicular complexes [45,59]. EVs are relevant not only as protective RNA carriers, but also because cargo packaging is selective and can reflect cellular state, stress responses, and tumor-microenvironment communication [60]. In bladder cancer, urine provides direct access to tumor-adjacent material and permits repeated low-burden sampling, while tissue anchors molecular findings to histology and blood provides a systemic perspective. These matrices are complementary but not interchangeable: an association observed in tumor tissue cannot be assumed to have the same direction, threshold, or clinical meaning in cell-free urine, urinary EVs, serum, or plasma.

### Clinical Positioning by Specimen and Intended Use

Urine, particularly cell-free urine and urinary EV fractions, is the most advanced matrix for non-invasive diagnostic development and serial NMIBC monitoring. Selected multi-lncRNA panels have functioned as stand-alone molecular classifiers within case–control development and internal-validation cohorts; however, their clinical role remains adjunctive because prospective multicenter studies have not established that cystoscopy or histopathology can be safely omitted [15,45,61,62,63]. Tumor-tissue lncRNAs are best positioned as adjuncts to pathological stage and grade, providing biological classification and prognostic refinement after transurethral resection of a bladder tumor (TURBT) or cystectomy rather than independent diagnosis [52,64,65,66,67,68,69]. Blood-based and circulating EV-associated lncRNAs may be informative in systemic or advanced disease and for molecular-response monitoring, but current evidence supports their use only within research or multivariable decision-support models [70,71,72,73]. Every clinical claim should therefore specify the specimen, molecular fraction, disease setting, endpoint, comparator, and intended decision. Tissue remains the reference matrix for linking lncRNA expression to histological subtype, grade, stage, and the tumor microenvironment. Measurement is feasible in TURBT and cystectomy specimens, including formalin-fixed paraffin-embedded (FFPE) material; however, fragmentation and chemical modification of FFPE-derived RNA make amplicon length, transcript region choice and quality control critical [74]. Tissue profiling can identify biologically coherent prognostic signals and treatment-associated pathways, but tissue cannot serve as a non-invasive screening test because it is obtained only after an invasive diagnostic or therapeutic procedure. Clinically relevant urine fractions include the pellet or sediment (enriched for exfoliated urothelial and inflammatory cells), cell-free urine (supernatant), and uEVs/exosomes, which may enrich tumor-derived RNA and reduce background [45,75]. Each captures different biology and requires a separate analytical validation strategy. Cellular RNA (urine pellet/sediment) may provide a strong cellular transcript signal when malignant cells exfoliate, but cell yield can fluctuate with tumor shedding, inflammation, hematuria, and collection conditions. uEV preparations can enrich protected, selectively packed RNA, but reported performance depends strongly on isolation and characterization methods. Limited reporting standardization remains a barrier to reproducibility and implementation [13,76]. The term exosome should be reserved for preparations in which small-EV biogenesis or appropriate subtype evidence supports that designation. Cell-associated RNA in the pellet more strongly reflects cellular composition and whole-cell transcriptional programs, whereas EV-enriched preparations may better reflect secreted or selectively packaged signals. Cell-free urine RNA offers workflow simplicity and scalability, but the signal may be dilute and is sensitive to hematuria burden, fragmentation, and inflammatory confounding. Exosomal noncoding RNAs (ncRNAs) in urine and blood have been suggested to have overall diagnostic promise for bladder cancer [13]. A recent meta-analysis of exosomal or EV-associated ncRNAs reported a pooled diagnostic sensitivity of 0.75 and specificity of 0.79 for bladder cancer detection, with summary areas under the receiver operating characteristic curve (AUCs) of approximately 0.83–0.84, with overall performance varying by RNA class and specimen type [13,76]. This reinforces that biomarker performance is influenced not only by the transcript selected, but also by the compartment and analytical pipeline used to measure it.

**Figure 1:**
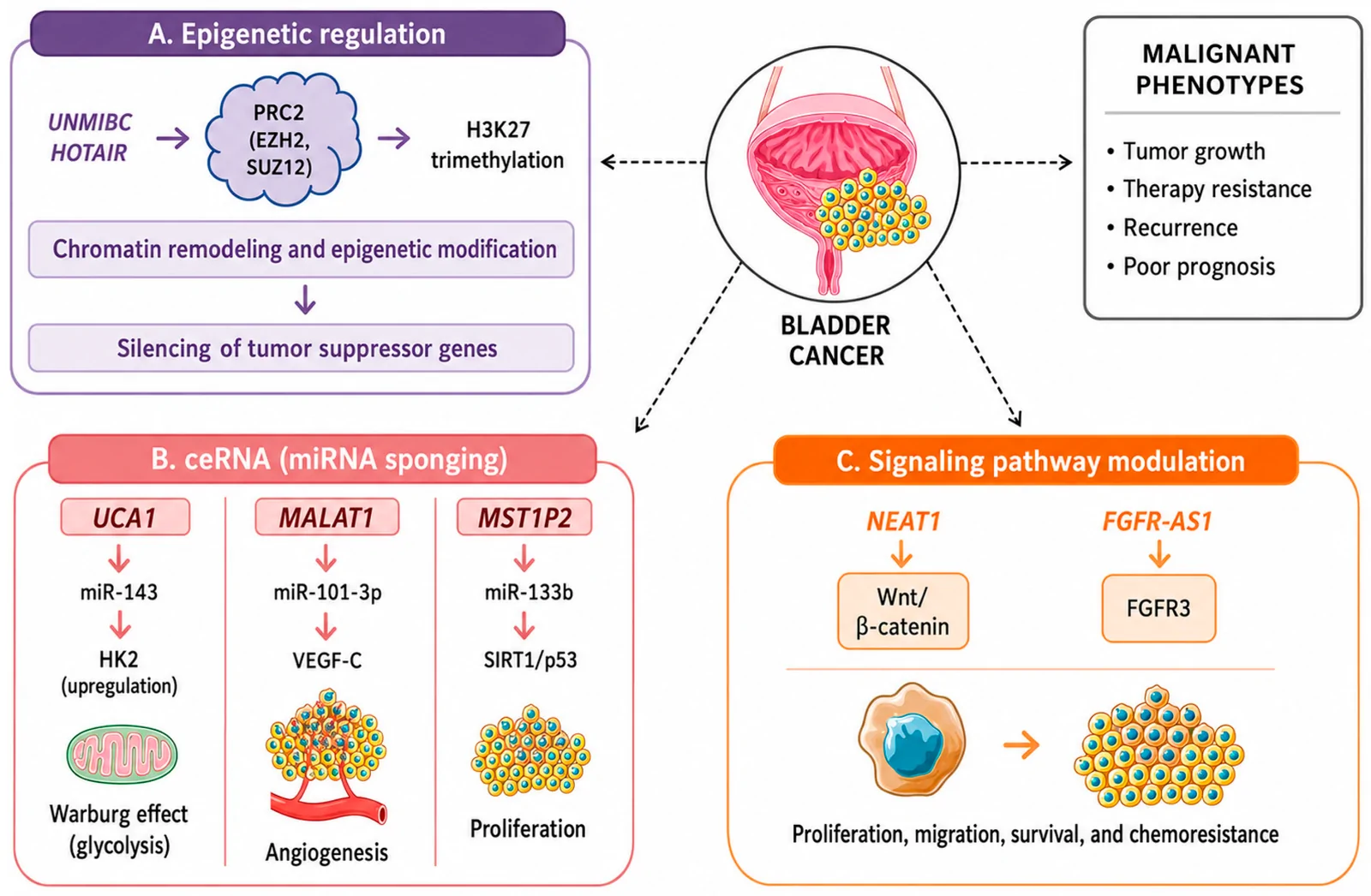
Mechanistic roles of selected lncRNAs in bladder cancer progression. Image provided by Servier Medical Art (https://smart.servier.com/), licensed under CC BY 4.0 (https://creativecommons.org/licenses/by/4.0/). (**A**) *HOTAIR* mediates PRC2-dependent H3K27 trimethylation and tumor suppressor genes silencing. (**B**) *UCA1, MALAT1,* and *MST1P2* act as miRNA sponges regulating glycolysis, angiogenesis or proliferation. (**C**) *NEAT1* and *FGFR-AS1* modulate Wnt/β-catenin and FGFR3 signaling. Together, these mechanisms drive malignant phenotypes. Abbreviations: ceRNA, competing endogenous RNA; FGFR3, fibroblast growth factor receptor 3; H3K27, histone H3 lysine 27; lncRNAs, long non-coding RNA; PRC2, polycomb repressive complex 2.

Blood offers a systemic window that may be particularly relevant to MIBC, metastatic disease, and treatment-associated monitoring. Candidate assays include cell-free RNA, circulating EVs/exosomal RNA, and less commonly, circulating tumor cells (CTCs)-associated lncRNA after circulating tumor cell enrichment [77,78]. A serum EV-associated three lncRNA model comprising *G023016, RP11-553N19.1,* and *LINC0087* derived from genome-wide microarray profiling has been evaluated for NMIBC detection and a serum exosomal *PCAT-1/UBC1/SNHG16* panel has been associated with diagnosis and recurrence risk [65,79]. Across all matrices, RNA sequencing is valuable for discovery because it enables unbiased detection, including isoforms and previously unannotated transcripts. For clinical deployment, however, targeted, multiplex assays (most commonly quantitative reverse transcription polymerase chain reaction [qRT-PCR]-based) are usually preferred because they are faster, cheaper per sample, and easier to standardize, and more compatible with defined decision thresholds [70]. The platform should therefore be selected based on the clinical use case (triage vs. risk stratification vs monitoring) and the compartment being measured, rather than by analytical convenience alone. Pre-analytical standardization is a decisive requirement for clinically reliable lncRNA biomarkers, particularly for EV/urinary assays where upstream variation can dominate measured differences. The urine fraction and processing workflow must be defined because pellet, supernatant and EV preparations contain different material, and centrifugation and isolation steps directly shape signal-to-noise and background composition [75]. Storage and handling also matter, as EV integrity and RNA yield can shift with temperature, buffer conditions, and freeze–thaw exposure, which can alter apparent transcript abundance and destabilize clinical cut-offs [80,81,82]. For blood-based assays, analogous attention is required for collection tubes, processing times, hemolysis, and storage. Hematuria and inflammation are major confounders because erythrocytes, leucocytes, infection and urinary inhibitors can alter apparent RNA abundance and amplification efficiency. Diagnostic performance should therefore be evaluated in consecutive referral cohorts containing benign hematuria, urinary infection, stones, recent instrumentation, and other realistic alternatives to cancer, rather than only healthy controls [83]. Normalization is another recurrent vulnerability. Reference targets may behave differently in sediment, cell-free urine and EV fractions, and classical housekeeping genes may not be stable across disease states or biofluid compartments. Empirical validation of endogenous controls, exogenous spike-ins, extraction efficiency, and analytical limits is essential. MIQE 2.0 emphasizes transparent reporting of pre-analytics, assay performance and normalization because small technical biases can materially affect low-abundance targets and clinical thresholds [70,84]. Finally, the reporting format should reflect the clinical decision being supported. A binary threshold may be appropriate for a prespecified rule-out or rule-in application, whereas a continuous score may better represent gradients of recurrence or progression risk. Diagnostic studies should report sensitivity, specificity, predictive values in the intended-prevalence population, likelihood ratios, calibration, and performance for high-grade disease and carcinoma in situ, rather than AUC alone. Prognostic studies should report adjusted effect estimates, calibration, discrimination, and clinical net benefit [15,45,80,85]. The mechanistic roles of selected lncRNAs in progression of bladder cancer are shown in Figure 1.

## 5. Urine-Based lncRNA Candidates and Panels—Diagnosis and Screening

Urine is an attractive substrate for bladder cancer biomarker testing because it is in direct contact with the urothelium and can contain exfoliated tumor cells, cell-free nucleic acids, and tumor-associated EVs/exosomes [86]. This proximity supports both diagnostic and surveillance-oriented testing [87], but urine is also a biologically heterogeneous inflammatory fluid [88]. Infection, stones, instrumentation, intravesical treatment, radiation cystitis and other benign urological conditions can alter RNA abundance and integrity [20]. Consequently, the clinically informative comparison is not cancer versus healthy volunteers, but cancer versus benign hematuria, inflammation, and the full spectrum of referral diagnoses [89]. Recent evidence syntheses support the feasibility of uEV and urine RNA markers in bladder cancer, while consistently highlighting heterogeneity, pre-analytical sensitivity, spectrum bias, and limited multicenter validation [13,76,90].

True population screening for bladder cancer remains challenging because prevalence is low, so even a test with apparently high sensitivity and specificity can generate many false-positive results [91]. The more realistic applications are hematuria triage, diagnostic prioritization, reflex testing after indeterminate cytology, and surveillance support in patients with established NMIBC [92]. A uEV lncRNA panel combined with NMP22 improved diagnostic value for bladder cancer and high-grade disease, illustrating the potential value of combining orthogonal signals [44]. Nevertheless, incremental AUC in a case–control cohort is not equivalent to clinical utility. Benefit must be demonstrated prospectively through safe reduction in low-yield procedures, earlier detection of clinically significant disease, or improved risk classification

Urinary lncRNA studies broadly fall into three practical categories: single lncRNA markers with replicated evidence, multi-lncRNA panels designed to stabilize performance across biological and technical variation and combined strategies where lncRNAs are assessed alongside cytology or established urine assays to increase clinical confidence. Across all three approaches, the evidentiary hierarchy should remain explicit. Analytical detectability and case–control discrimination are early developmental steps; independent external validation, locked thresholds, prospective recruitment, and pathway-level utility are required before clinical adoption.

### 5.1. Single lncRNA Urine Markers with Replicated Evidence

Among urine-based lncRNAs, UCA1 is the most frequently examined single marker across different analytical formats and clinical settings. Its diagnostic plausibility is supported by repeated detection in urine and by experimental evidence that *UCA1* can promote glycolysis, proliferation, survival, and drug resistance through several context-dependent pathways, including miR-143/HK2-related metabolic regulation [93]. Urinary *UCA1* has been evaluated using amplification-based assays, nanoparticle hybridization, and RNA in situ hybridization applied to cytology preparations [94,95,96]. In a recent cytology-based study, adding *UCA1* improved sensitivity and negative predictive value for high-grade disease [95]. These findings support *UCA1* as an adjunctive urinary marker, but assay platforms, transcript targets, control groups, and thresholds differ substantially; a universal *UCA1* cut-off cannot be transferred across studies. The apparently discordant *UCA1* findings are biologically and clinically reconcilable. Experimental comparisons generally show that increased *UCA1* relative to non-malignant urothelium supports oncogenic phenotypes. By contrast, the clinically annotated study by Avgeris et al. [97] evaluated variation within bladder tumors and found that lower tumor-tissue *UCA1* expression was associated with higher stage and grade and, among patients with NMIBC, with increased risks of early recurrence and progression. Between-group diagnostic discrimination and within-tumor prognostic stratification are different questions and need not show the same direction. Additional contributors may include disease stage, cellular differentiation, transcript isoforms, tumor-cell composition, and matrix-specific release or packaging, although these explanations require direct validation. Clinically, *UCA1* should therefore be interpreted only within a validated matrix-, transcript-, assay-, and endpoint-specific model; urinary elevation cannot be assumed to carry the same prognostic meaning as tumor-tissue expression.

Other single-candidate uEV-associated lncRNAs include *TERC, SNHG16*, and *SUMO1P3*. *TERC* (the telomerase RNA component) aligns with telomerase-driven replicative immortality [98,99] and has been reported in urinary exosomes with diagnostic performance exceeding NMP22 and cytology [85]. Urinary exosomal *TERC* showed sensitivity of 78.65% and overall accuracy of 77.78% [85]. Also, small nucleolar RNA host gene 16 (*SNHG16*) has been measured in urinary exosomes and enabled diagnostic discrimination between cancer and controls in a case–control design, with performance reported as superior to cytology in that cohort. Urinary exosomal *SNHG16* achieved an AUC of 0.791, sensitivity of 61.9%, and specificity of 83.3% in a cohort of 42 patients and 42 controls, compared with an AUC of 0.597 for cytology [16]. Mechanistically, it has been linked to bladder cancer progression via a miR-98/STAT3/Wnt/β-catenin axis, providing some biological plausibility alongside statistical separation [16]. Finally, the small ubiquitin modifier 1 pseudogene 3 (*SUMO1P3*) has recently emerged as a urine candidate marker associated with aggressive phenotypes. In a retrospective cohort, *SUMO1P3* increased with higher-risk disease states (high-grade NMIBC and MIBC). It was explored together with its interacting microRNA (miR-320a) as a progression-related signal, providing a mechanistic narrative (RNA–RNA interaction) [100]. These results remain exploratory: single-marker estimates are especially susceptible to center effects, inflammatory confounding, threshold optimization, and small-sample uncertainty, and none has yet been validated as an independent clinical rule-out or surveillance test. In hematuria populations, cystitis- or infection-driven false positives are common rather than exceptional, and this is why single-marker performance often drops when moving from case–control studies to true referral cohorts [18,42,101].

### 5.2. Multi-lncRNA Panels and Combined Strategies

Multi-marker modeling is biologically attractive because bladder cancer is heterogeneous with respect to grade, stage, molecular subtype, tumor burden, and RNA release, while urine composition varies with inflammation and benign disease. The *MALAT1/PCAT-1/SPRY4-IT1* urinary exosome panel was developed in 104 patients and 104 healthy controls and evaluated in an internal validation cohort of 80 patients and 80 controls [102]. The reported areas under the receiver-operating characteristic (ROC) curves (AUCs) were 0.854 and 0.813, respectively, compared with 0.619 for cytology. The proposed biology of these lncRNAs is consistent with a “signal enrichment” of malignant program, including epithelial–mesenchymal transition (EMT), proliferation and epigenetic regulation. *MALAT1* promotes EMT and metastatic traits in bladder cancer (including Wnt-associated EMT programs), *PCAT-1* supports proliferation and survival (silencing induces growth arrest and apoptosis). *SPRY4-IT1* can act as a competing endogenous RNA that increases EZH2 by sponging miR-101-3p, thereby facilitating proliferation and metastasis [61,102,103,104]. Higher exosomal *PCAT-1* and *MALAT1* were also associated with poorer recurrence-free survival. The study established proof of concept, but its healthy-control, single-system case–control design limits direct inference for hematuria clinics and NMIBC surveillance. A four-lncRNA cell-free urine panel comprising *UCA1-201*, *HOTAIR, HYMA1* and *MALAT1* was developed specifically to distinguish bladder cancer from chronic urocystitis, a more clinically relevant comparator than healthy status [105]. In a study of 140 patients with NMIBC and 140 with urocystitis, the panel achieved sensitivity of 95.7% and specificity of 94.3%. Applicability was subsequently assessed in an independent set of 60 patients per group. This design addresses inflammatory confounding more directly, but it remains a single-center case–control study and does not establish performance across the broader hematuria spectrum, upper-tract malignancy, or surveillance after intravesical treatment. One component of this panel, *HOTAIR*, is associated with bladder cancer progression and is detectable in patients’ urinary exosomes, where it contributes to invasive behavior. The knockdown of this lncRNA has been demonstrated to reduce migration/invasion in model systems [62]. Compartment-aware panel development is another important direction because pellet/sediment RNA and EV RNA are not interchangeable. A panel comprising *RMRP, UCA1*, and *MALAT1* generated an EV-based support-vector-machine classifier with an AUC of 0.875, sensitivity of 80.0%, and specificity of 81.4%. Mechanistic studies indicate that *RMRP* promotes bladder cancer cell proliferation, migration, and invasion. More recent work showed that m6A-stabilized *RMRP* epigenetically suppresses the tumor suppressor *SCARA5* through promoter methylation, supporting its inclusion as a biologically grounded rather than purely statistical panel feature [63,106]. Similarly, a two-lncRNA cell-free urine signature based on uc004cox.4 + GAS5 was developed using microarray discovery and evaluated in large training and validation sets. It achieved an overall AUC of 0.885 that exceeded urine cytology across stages and stage-specific AUCs of 0.843 for Ta, 0.867 for T1, and 0.923 for T2–T4, with an observational association with recurrence-free survival [107]. These studies support the feasibility of fraction-specific signatures, but small or selected case–control cohorts, retrospective outcome analyses, and limited external validation leave uncertainty about calibration and clinical net benefit. More recently, multi-phase studies have expanded the panel landscape. Li et al. [15] reported a three-lncRNA urine exosome “fingerprint” (*CCDC148-AS1, XLOC_006419, RP5-1148A21.3*) that was verified across several phases including a cohort of 200 NMIBC participants. The signature was reported as superior to urinary cytology for discriminating NMIBC from healthy controls. Similarly, Chen et al. [44] identified a urinary EV lncRNA panel comprising *MALAT1, SCARNA10, LINC00963*, and *LINC01578*, which showed improved discrimination when combined with NMP22, including high-grade disease (reported AUCs: 0.917). Such results are promising, particularly when high-grade disease is reported separately, but recent single-system evidence requires independent multicenter replication with prespecified processing, normalization, thresholds, and clinically representative controls. lncRNA panels may be most useful when they complement, rather than duplicate, established urine tests and cytology. Clinically informative panel studies include patients with non-cancer causes of hematuria, such as cystitis and urinary stones. They also report performance separately for high-grade tumors and CIS; otherwise, the results may not reflect how well the test will perform in hematuria clinics or bladder cancer surveillance [108,109,110]. The principal barriers to urinary lncRNA implementation are now as much analytical as biological. Collection time, urine volume and concentration, hematuria, bacterial contamination, storage, freeze–thaw exposure, centrifugation, EV isolation, RNA extraction, and normalization can all change the measured signal. Sediment, cell-free supernatant, and urinary EV preparations should not be pooled conceptually or analytically. Rapid platforms, including RT-RAA-CRISPR/Cas12a detection of exosomal *RMRP*, may improve turnaround and laboratory fit, but speed does not remove the need for analytical validation, contamination controls, reproducibility studies, and prospective assessment in the intended clinical pathway [106,111].

## 6. lncRNAs for Prognostic Risk Stratification, Recurrence, and Progression

lncRNAs are being investigated as prognostic biomarkers that may refine risk beyond stage, grade, tumor size, multiplicity, concomitant carcinoma in situ, and established clinical scores. The need is particularly acute in NMIBC, where recurrence is common, but progression risk is heterogeneous. A clinically useful prognostic assay must provide information beyond standard variables, use a prespecified cut-off, retain performance after multivariable adjustment, and demonstrate calibration and external validity. Many published studies meet only part of this standard. Associations from small retrospective cohorts should therefore be interpreted as candidate prognostic signals rather than practice-ready risk tools [112]. In NMIBC, tissue-based lncRNAs associated with chromatin regulation provide a clear example of biologically anchored prognostic biomarkers. *HOTAIR/EZH2* has been repeatedly connected to aggressive behavior and recurrence. It is consistent with the concept that lncRNAs can amplify residual malignant potential even when routine features appear favorable [64,113]. Martínez-Fernández et al. [113] reported that gene sets regulated by the *HOTAIR-EZH2* axis could effectively distinguish non-tumoral samples from bladder cancer and could further separate recurrent from non-recurrent disease. This study also linked *HOTAIR* to polycomb-associated epigenetic regulation, providing a biologically plausible explanation for the association between its expression and clinical outcomes. From a risk-modeling perspective, single-marker studies provide stronger prognostic evidence when the marker remains associated with outcome after adjustment for established clinicopathological factors, rather than only in unadjusted analyses. The *UCA1* program incorporated internal validation and formally evaluated clinical utility using decision-curve analysis [97,112]. In that study, lower *UCA1* expression was linked to more aggressive disease, including MIBC and higher stage and grade. In patients with NMIBC, decreased *UCA1* levels were also associated with worse short-term outcomes, showing an increased risk of early recurrence (HR 1.974; *p* = 0.032) and a higher likelihood of progression to invasive disease (HR 3.476; *p* = 0.023) [97]. These examples collectively indicate that lncRNAs can add incremental prognostic information, but independent external validation using the same transcript target and assay is required before clinical use. Serial liquid-based lncRNA testing is attractive because it could capture changing biological risk after TURBT. A urinary EV lncRNA panel comprising *MALAT1*, *PCAT-1* and *SPRY4-IT1* showed both diagnostic and prognostic potential in bladder cancer [102]. In patients with NMIBC, higher exosomal *PCAT-1* and *MALAT1* expression was associated with shorter recurrence-free survival, although only *PCAT-1* remained independently associated with recurrence in multivariable analysis [102]. Complementary blood-based evidence has also been reported. A serum exosomal panel including *PCAT-1/UBC1/SNHG16* demonstrated diagnostic value, while high UBC1 expression was independently associated with shorter recurrence-free survival in NMIBC [65]. These findings may support the feasibility of liquid-based lncRNAs as adjunctive prognostic biomarkers, but they do not establish that such assays can safely determine surveillance intervals or replace cystoscopy. In MIBC, in which outcomes are currently driven by occult spread and early relapse, transcriptome-derived lncRNA signatures have been investigated for postoperative recurrence and survival prediction [71]. An eight-lncRNA model developed using 320 cases from The Cancer Genome Atlas stratified disease-free survival and achieved a concordance C-index of 0.719 [71]. However, the model was evaluated through an internal split of the same cohort rather than in an independent external population. Individual tissue biomarkers have also shown prognostic associations. In a cohort of 98 patients with MIBC, high *PVT1* expression remained independently associated with poorer overall survival (HR 2.381, 95% CI 1.821–7.012; *p* = 0.014) [66]. Large-scale molecular profiling further indicates that lncRNA expression reflects biologically relevant tumor heterogeneity. TCGA analysis identified lncRNA-defined subgroups associated with distinct molecular characteristics and survival outcomes [67]. Subsequent studies identified a luminal-favorable subtype characterized by organ-confined, node-negative disease, increased FGFR3 pathway activity, and improved survival after radical cystectomy [68]. These classifiers appear prognostic, but current evidence does not demonstrate that they predict benefit from neoadjuvant chemotherapy or another specific treatment. Overall, liquid- and tissue-based lncRNAs may provide prognostic information beyond conventional clinicopathological factors, but their clinical implementation requires prospective validation using standardized assays, prespecified cut-offs, and independent cohorts. Longitudinal studies should also determine whether serial urine or blood lncRNA measurements provide clinically meaningful lead time over cystoscopy or imaging. Until such evidence is available, these markers should be regarded as candidate adjunctive tools rather than validated surrogates of minimal residual disease [65,102].

## 7. Treatment-Associated lncRNAs and Candidate Predictive Biomarkers

Treatment guidance requires a stricter evidentiary standard than prognosis. A biomarker associated with non-response or poor survival in a treated cohort may simply identify aggressive disease. A genuinely predictive biomarker demonstrates that the relative benefit or harm of a specified treatment differs according to biomarker status, ideally through a prespecified biomarker-by-treatment interaction in a prospective comparative or randomized study. By this standard, the available bladder-cancer lncRNA literature is predominantly mechanistic, treatment-associated, and hypothesis-generating. In NMIBC treated with intravesical BCG, early identification of BCG-unresponsive disease could prevent ineffective retreatment and delayed cystectomy. *MIR4435-2HG* was identified as a BCG-response-associated lncRNA through analyses of public transcriptomic datasets and was also linked to prognosis [69]. This study suggests a biologically relevant candidate, but it does not establish a validated predictive test since it was computational and retrospective, treatment definitions and sampling were not designed for prospective clinical classification, and differential benefit from BCG versus an alternative treatment was not tested. Validation requires uniformly treated contemporary cohorts, accepted BCG-unresponsive definitions, locked assays, and comparative or interaction-based analyses [114]. Dynamic molecular measurements may still be useful for response monitoring even when they are not predictive [115]. Serum exosomal *BCYRN1* levels fall after complete surgical removal of bladder tumors while failure to decline in an illustrative patient preceded residual tumor and later recurrence [115]. Patients who showed this post-operative decline remained recurrence-free for over two years after surgery. This observation supports biological response tracking but remains insufficient to define a surveillance threshold or to predict benefit from a specific therapy [115]. The same distinction applies to urinary EV signatures—feasibility of serial measurement does not by itself establish clinical utility.

In *MIBC, MST1P2, UCA1, MALAT1, NEAT1*, and *LINC00857* have been linked to platinum resistance through apoptosis, DNA-damage, metabolic, and stress-response pathways [72,116,117,118,119]. In this cancer, the clinical relevance of a treatment biomarker depends on whether it can identify patients who are more likely to benefit from a specific therapy, rather than merely identifying biologically aggressive disease. This distinction is particularly important for perioperative cisplatin-based neoadjuvant/adjuvant chemotherapy (NAC/AC) for which the expected therapeutic gain must be balanced against toxicity, renal eligibility and the risk of postponing definitive local therapy. Experimental studies indicate that several lncRNAs participate in cellular pathways involved in platinum resistance, including apoptosis regulation, DNA-damage signaling, stress adaptation, and prosurvival signaling. These observations support biological plausibility, but they are insufficient to establish treatment-specific predictive utility [72]. For example, *MST1P2* provides one example of a mechanistically characterized resistance-associated lncRNA. Its expression is increased in cisplatin-resistant bladder cancer cells, where it modulates the *miR-133b*/SIRT1/p53 regulatory network and thereby promotes resistance to cisplatin [72]. *LINC00857* has been examined in both treatment-annotated datasets and experimental systems. Higher expression was observed in patients with poor response to platinum-based chemotherapy, whereas suppression of *LINC00857* increased cisplatin sensitivity through an LMAN1-related mechanism [116]. However, the retrospective nature of the clinical observations prevents firm conclusions regarding treatment-specific survival benefit. Other lncRNAs have been linked to platinum resistance almost exclusively through preclinical models. *UCA1* influences cisplatin sensitivity through WNT6-associated Wnt signaling, while *MALAT1* modulates resistance through interactions involving *miR-101-3p* and VEGF-C [117,118]. Increased *NEAT1* expression has also been observed in cisplatin-resistant bladder cancer cells; experimental silencing of *NEAT1* reduced proliferation and invasiveness and enhanced apoptosis after cisplatin exposure [119]. Collectively, these studies identify molecular processes that may contribute to treatment resistance, but they do not demonstrate that measuring these transcripts can guide chemotherapy selection in clinical practice. A clinically useful predictive biomarker must be evaluated differently from a general prognostic marker. Studies should account for tumor stage, renal function, performance status, treatment intensity, and other variables that influence both treatment allocation and outcome. Whenever possible, the analysis should include a formal interaction between biomarker status and treatment exposure. Prospective or randomized cohorts are preferable because retrospective comparisons cannot reliably distinguish treatment prediction from baseline prognostic differences. Moreover, a biomarker proposed for perioperative decision-making should ultimately identify an improvement in clinically meaningful endpoints, such as overall or bladder cancer-specific survival, rather than only an association with pathological response [120,121].

lncRNAs have also been investigated in relation to radiotherapy. Tumor response to radiation is influenced by DNA-repair capacity, hypoxia, epithelial–mesenchymal plasticity, and cellular stress responses. Experimental inhibition of *TUG1* increased radiosensitivity in bladder cancer models through reduced HMGB1 activity [122,123]. A separate 10-lncRNA model, initially derived from cell-line radiosensitivity data, was subsequently examined in 151 patients with MIBC treated with radiotherapy. The classifier-defined radioresistant group had less favorable local relapse-free survival, but the multivariable association did not reach conventional statistical significance (*p* = 0.065) [123]. The model should therefore be considered exploratory rather than suitable for treatment allocation. Hypoxia-associated lncRNA signatures have similarly been linked to survival, immune composition, and computational estimates of therapeutic response, but most have been developed from retrospective transcriptomic datasets and remain unvalidated in prospective radiotherapy cohorts [124,125].

For immune checkpoint inhibitors, the strongest biological rationale concerns lncRNAs involved in interferon signaling, antigen processing, immune cell recruitment, T-cell dysfunction, and checkpoint regulation because these pathways sit upstream of PD-1/PD-L1 biology and response heterogeneity [126,127]. Interferon-Stimulated Noncoding RNA 1 (*INCR1*) is transcribed from the PD-L1 locus, was shown to modulate interferon-gamma (IFNγ)-responsive genes, including PD-L1 and JAK2, through interaction with HNRNPH1 [128]. Although this mechanism is relevant to immune regulation, *INCR1* has not been established as a clinically useful bladder cancer biomarker. Likewise, several immune-related lncRNA signatures have been associated with immune-cell infiltration and overall survival and have been assessed using TIDE-based estimates of immunotherapy responsiveness [126,129]. Such computational scores generate hypotheses about immune escape but do not provide direct evidence of benefit among patients treated with checkpoint inhibitors. More clinically direct evidence has been reported for EV-associated MAFG antisense RNA 1 (*MAFG-AS1*). In a cohort of 52 patients with advanced urothelial carcinoma receiving pembrolizumab, circulating EV-derived *MAFG-AS1* was associated with treatment response and survival outcomes [130]. This treatment-annotated design provides stronger evidence than purely computational modeling. However, the cohort was small, and the result requires confirmation in independent prospective studies using prespecified analytical thresholds and adjustment for established prognostic factors.

In advanced urothelial carcinoma, lncRNAs may ultimately be more useful as markers of tumor state or pathway activity than as independent selectors of targeted therapy. FGFR3 antisense RNA 1 (*FGFR3-AS1*) is overexpressed in bladder cancer, and its experimental inhibition reduces proliferation and motility while increasing apoptosis, indicating a relationship with FGFR3-associated tumor biology [73,131]. Current selection for FGFR-directed therapy, however, remains based on qualifying FGFR2/3 genomic alterations, and no lncRNA assay has demonstrated additional predictive value. Similarly, although enfortumab vedotin acts through NECTIN4, there is no validated lncRNA-based strategy for selecting patients for this treatment. Transcriptomic markers may eventually help define lineage state, target heterogeneity, or resistance mechanisms, but the available evidence is currently insufficient [132,133,134,135].

lncRNAs are also being explored as therapeutic targets. Bladder cancer offers a potentially favorable setting for local nucleic-acid delivery because agents can be administered intravesically. In experimental models, CRISPR/Cas9-mediated disruption of *UCA1* together with modulation of the PD-1 pathway produced enhanced antitumor effects [136,137,138,139]. These findings remain preclinical and should not be presented as evidence of therapeutic efficacy in patients. Translation will require reliable delivery, selective targeting of relevant transcripts and isoforms, adequate mucosal penetration, molecular stability, and careful control of off-target and immune effects. Table 1 summarizes the principal lncRNA panels investigated for bladder cancer diagnosis and prognosis.

## 8. Integrating lncRNAs into Clinical Pathways

Integrating lncRNAs into clinical pathways is most realistic when they are treated as decision-support tools that complement clinical risk factors, imaging and cytology, and guideline-directed cystoscopy [140]. Clinical adoption requires an explicit intended-use statement defining the target population, specimen, position in the pathway, decision threshold, comparator, and action triggered by a positive, negative, or indeterminate result [108,141]. In hematuria pathways, a urinary lncRNA panel could be evaluated as a triage add-on after standard risk assessment, with the aim of prioritizing patients at higher molecular risk and reducing low-yield procedures in carefully selected low-risk patients. No current lncRNA result should be used to defer an otherwise indicated cystoscopy outside a prospectively validated protocol [20,45,142]. In NMIBC surveillance, serial urinary lncRNA measurements could be evaluated between guideline-scheduled cystoscopies as an early-warning or reassurance signal. Persistently negative results might eventually support protocolized de-intensification in selected patients, whereas a newly positive or rising score might prompt earlier cystoscopy or enhanced imaging. Such use requires prospective non-inferiority or safety-oriented trials with high-grade recurrence and carcinoma in situ as explicit outcomes. Observational associations, including those reported for *SUMO1P3*, are insufficient to define surveillance intervals or action thresholds [100]. For MIBC, where cisplatin-based neoadjuvant chemotherapy, immunotherapy strategies in appropriate settings and bladder-preserving trimodality therapy each carry distinct benefit–toxicity trade-offs, the most defensible near-term role is incorporation of lncRNAs into validated multivariable models for prognosis or treatment-associated outcomes, not independent treatment selection. Any model must demonstrate incremental value beyond stage, grade, imaging, performance status, renal function, molecular subtype, and established treatment biomarkers, and must be tested in a cohort representative of the clinical decision under consideration [35,143,144]. Clinical-grade evidence requires prospective recruitment, prespecified endpoints, locked assays and cut-offs, transparent handling of indeterminate results, and within-patient comparison with standard practice. Diagnostic outcomes should include sensitivity for high-grade disease and carcinoma in situ, predictive values at the intended prevalence, procedures avoided, diagnostic delay, and patient-reported burden. Prognostic and monitoring studies should include recurrence, progression, calibration, decision-analytic net benefit, and clinically meaningful lead time. The STRATA randomized trial of a different urine-test-guided strategy illustrates the pathway-level standard: procedural reduction accompanied by formal safety assessment [145]. lncRNA-based assays should meet comparable evidentiary and health-economic standards before routine implementation. The biological pathways and clinical relevance of selected lncRNAs are summarized in Table 2.

## 9. Limitations, Gaps, and Future Perspectives

The biological rationale for lncRNA biomarkers in urothelial carcinoma is strong, but the clinical evidence remains limited by generalizability, comparability, and decision relevance. First, most urinary lncRNA panels were developed in single-center or single-system case–control cohorts, frequently using healthy volunteers rather than consecutive patients with hematuria or previous bladder cancer. This design inflates apparent separation, limits estimation of predictive values, and creates spectrum bias. Heterogeneous collection, processing, EV isolation, RNA extraction, transcript definitions, and normalization further impede direct comparison and may explain part of the between-study variability reported in meta-analyses [15,44]. Second, there is still a shortage of head-to-head comparisons. New lncRNA assays are not consistently compared with cytology, established urine tests, clinical risk models, and cystoscopy in the same patients using prespecified thresholds. Consequently, it is often unclear whether a panel adds independent information or merely reflects differences in recruitment and sample handling [23,44]. High AUCs from internally validated case–control studies should not be interpreted as proof of clinical utility. Utility requires demonstration that testing changes management and improves a meaningful outcome without an unacceptable increase in missed high-grade disease, false-positive investigation, cost, or burden. Third, longitudinal evidence is particularly sparse. Cross-sectional diagnostic designs cannot determine whether serial lncRNA changes anticipate recurrence, whether a negative result remains reassuring over time, or what molecular change should trigger earlier cystoscopy. Monitoring candidates such as *SUMO1P3* and *BCYRN1* require protocolized sampling before and after treatment, blinded outcome ascertainment, time-dependent modeling, and validation of clinically actionable lead times [100,115]. Treatment-associated studies face additional confounding by indication and baseline risk; without comparative treatment groups and interaction testing, they cannot establish prediction. Finally, real-world confounding must be incorporated deliberately. Common urological states, such as UTI, stones, visible or microscopic hematuria, recent catheterization or cystoscopy, intravesical therapy, renal function, and inflammatory bladder disorders, may alter cell composition, EV abundance, RNA integrity, or amplification [23,45,146]. These variables should be prespecified, measured, and either modeled or used in sensitivity analyses. EV studies should follow MISEV2023 consensus principles for isolation and characterization and qPCR studies should report MIQE 2.0-relevant analytical information, including extraction controls, transcript identity, amplification efficiency, limits of detection, and reference-target stability [80,142,146].

The immediate priority is prospective, multicenter validation in three clinically distinct settings: consecutive hematuria referrals undergoing complete diagnostic work-up, NMIBC surveillance cohorts with protocolized cystoscopy and urine collection, and treatment-annotated BCG chemotherapy, radiotherapy, or immunotherapy cohorts with contemporary endpoint definitions. The target population, clinical decision, primary endpoint, and acceptable error rates should be specified before assay evaluation. Diagnostic studies should enrich neither cases nor controls artificially and should report high-grade and carcinoma-in situ performance separately. Prognostic studies should use external validation and decision-curve analysis. Predictive studies should include a relevant treatment comparator and formal interaction testing whenever feasible. Composite models are likely to be more clinically useful than isolated transcripts. lncRNA signals can be combined with age, smoking exposure, prior tumor characteristics, cytology, imaging, DNA alterations, or protein markers such as NMP22, but added complexity must be justified by calibration, reclassification, net benefit, cost, and reproducibility [45]. Transferability requires harmonized protocols for urine fraction, collection timing, stabilization, centrifugation or EV isolation, storage, RNA extraction, quality control, and normalization. Progress should now be judged less by the number of newly reported lncRNAs than by successful external validation, transparent assay lock-down, and evidence that lncRNA-informed pathway safely improves patient care.

## 10. Conclusions

lncRNAs are biologically credible biomarkers in bladder cancer because they can reflect tumor biology, but their clinical interpretation is inseparable from specimen type, molecular fraction, disease setting, endpoint, and evidence quality. Diagnostic, prognostic, treatment-associated, and predictive claims are not interchangeable. No lncRNA assay is currently validated as a stand-alone replacement for cystoscopy, cytology, histopathology, or established molecular treatment-selection tests. The most mature translational evidence concerns urine-based assays. Multi-lncRNA signatures measured in cell-free urine or urinary EVs can function as statistical classifiers in development studies, and panels tested against urocystitis or combined with established urine markers are particularly informative. However, the predominantly single-center case–control evidence is vulnerable to spectrum bias, threshold optimization, and pre-analytical variability. The appropriate near-term role is decision support in hematuria triage, reflex testing after equivocal cytology, and serial surveillance research alongside guideline-directed cystoscopy. Tumor-tissue lncRNAs may refine prognostic assessment after TURBT or cystectomy, whereas circulating lncRNAs may complement evaluation of systemic disease and molecular response. *UCA1* exemplifies the importance of context. Increased expression promotes oncogenic phenotypes in experimental systems and can support urinary detection, whereas lower tumor-tissue expression within a selected NMIBC cohort was associated with early recurrence and progression. These observations concern different comparisons and do not support a universal high-versus-low interpretation. Clinical use requires a validated transcript or isoform, specimen fraction, assay, normalization strategy, endpoint, and cut-off. Treatment-guidance evidence is substantially less mature. Most studies are mechanistic, retrospective, computational, or based on response within a single treated cohort and should not be labeled predictive without comparative treatment data and biomarker-by-treatment interaction testing. The decisive next steps are prospective multicenter studies in representative hematuria, surveillance, and treatment-annotated populations; direct within-patient comparison with standard tests; harmonized pre-analytics and normalization; locked thresholds; external validation; and reporting of calibration, clinical net benefit, high-grade disease, carcinoma in situ, recurrence, progression, treatment benefit, and patient-level outcomes

## Figures and Tables

**Table 1:** Structured evidence-quality appraisal of representative lncRNA biomarker-panel studies.

Panel/Marker	Study Design and Sample Size	Biological Material/ Model	Assay and Normalization	Validation	Main Findings	Major Limitations	Ref.
*MALAT1, PCAT-1, SPRY4-IT1*	Case–control development and internal validation: training 104 BC/104 controls; validation 80/80	Urinary EVs described as exosomes	qRT-PCR; relative-expression normalization as reported	Separate internal validation cohort	AUC 0.854 (training) and 0.813 (validation); cytology AUC 0.619; recurrence associations reported	Healthy controls; single-system case–control design; limited clinical spectrum; prognostic analyses exploratory	[102]
*UCA1-201,* *HOTAIR, HYMA1, MALAT1*	Case–control development: 140 NMIBC/140 urocystitis; independent evaluation: 60/60	Urinary cell-free RNA	qRT-PCR; GAPDH; relative quantification	Independent cohort within the same center	Sensitivity 95.7% and specificity 94.3% in development; applicability confirmed in 60/60 validation cohort	Single center; NMIBC versus urocystitis only; performance across broader hematuria and surveillance populations unknown	[105]
*RMRP, UCA1, MALAT1*	Case–control: training 22 BC/20 controls; validation 33/23	Urinary EVs and urine sediment	qRT-PCR; GAPDH-based relative quantification	Separate validation cohort; SVM classifier	EV-based SVM AUC 0.875; sensitivity 80.0%; specificity 81.4%	Small cohorts; healthy controls; single center; no longitudinal clinical-utility validation	[106]
*uc004cox.4 + GAS5*	Microarray discovery followed by qRT-PCR training/validation; qRT-PCR cohort 110 BC/110 controls	Urinary cell-free RNA	qRT-PCR; GAPDH; relative quantification	Training and validation sets	Overall AUC 0.885; stage-specific AUCs 0.843 (Ta), 0.867 (T1), and 0.923 (T2-T4); recurrence association reported	Case–control spectrum; retrospective prognostic analysis; multicenter external validation not established	[107]
*CCDC148-AS1, XLOC_006419, RP5-1148A21.3*	Multiphase study: microarray discovery 10 NMIBC/10 healthy controls; verification phases included 200 NMIBC participants	Urinary EVs described as exosomes	Microarray followed by qRT-PCR; normalization NR	Multiphase internal verification	AUC up to 0.873; reported to outperform cytology	Healthy-control comparison; single-system development; no prospective pathway-level utility study	[15]
*MALAT1, SCARNA10, LINC00963, LINC01578 ± NMP22*	RNA-sequencing discovery with qRT-PCR evaluation; sample allocation NR	Urinary EVs	RNA sequencing followed by qRT-PCR; normalization NR	Development and verification reported; independent external validation not established	AUC 0.900 for BC; AUC 0.917 for high-grade disease when combined with *NMP22*	Recent single-system evidence; incomplete external validation; no prospective multicenter clinical-utility trial	[44]
*G023016, RP11-553N19.1, LINC0087*	Serum-EV discovery with training and expanded validation; sample allocation NR	Serum EVs described as exosomes	Genome-wide microarray followed by targeted validation; normalization NR	Training and expanded validation within the study	AUC 0.827 in training and 0.809 in expanded validation	Blood matrix; case–control design; intended-use population and external generalizability require confirmation	[79]
*PCAT-1, UBC1, SNHG16*	Case–control: 160 BC/160 controls; observational recurrence analysis	Serum EVs described as exosomes	qRT-PCR; normalization NR	Training and validation sets within one study	Panel AUC 0.857 (training) and 0.826 (validation); *UBC1* associated with recurrence risk	Single-system case–control evidence; serum matrix; recurrence association observational and not treatment-predictive	[65]

Abbreviations: AUC, area under the receiver-operating-characteristic curve; BC, bladder cancer; EV, extracellular vesicle; NMIBC, non-muscle-invasive bladder cancer; NR, not reported; qRT-PCR, quantitative reverse-transcription polymerase chain reaction; SVM, support-vector machine.

**Table 2:** Long non-coding RNAs in bladder cancer: mechanisms and clinical relevance.

lncRNA	Biological Material/Model	Expression	Mechanism	Functional Role/Clinical Relevance	Ref.
*UNMIBC*	NMIBC tissues; BC cell lines; xenograft model	↑	PRC2/EZH2-mediated epigenetic regulation	Promotes tumor growth in experimental models; associated with recurrence in tissue cohorts	[51]
*UCA1*	BC cell lines; tumor tissue; urine	Context-dependent; often ↑ vs. non-malignant urothelium	ceRNA activity via *miR-143* and regulation of glycolysis; other context-dependent pathways	Promotes glycolysis (Warburg effect) and proliferation; in experimental models; diagnostic and prognostic direction varies by specimen, cohort, assay, and disease context	[93]
*TERC*	Urinary exosomes (BC vs. controls)	↑	Telomerase-associated regulation (*TERC/TERT* axis)	Candidate urinary diagnostic marker; prognostic utility requires validation	[85]
*SNHG16*	Urinary exosomes (BC vs. healthy); BC cell lines	↑	*miR-98*/STAT3/Wnt/β-catenin-related signaling	Tumor-promoting, candidate diagnostic marker supported mainly by case–control evidence	[16]
*SUMO1P3*	Urine samples (NMIBC/MIBC)	↑	miRNA-mediated regulatory axis (miR-320a)	Associated with higher-risk disease and progression; surveillance utility not established	[100]
*HOTAIR*	NMIBC/MIBC tissues; TCGA datasets; BC cell lines	↑	PRC2/EZH2-mediated epigenetic regulation	Associated with recurrence and poor overall survival; evidence predominantly retrospective	[113]
*PVT1*	MIBC tissues	↑	Not fully elucidated	Associated with advanced stage, metastasis, and poor prognosis; external clinical validation limited	[66]
*MIR4435-2HG*	TCGA and MiTranscriptome datasets; NMIBC/MIBC cohorts	↑	Not fully elucidated	Prognostic and BCG-response-associated candidate; differential treatment benefit not demonstrated	[69]
*BCYRN1*	BC cell lines; serum exosomes	↑	Cell cycle–associated regulation	Promotes proliferation and invasion; dynamic serum-EV changes are an exploratory marker of tumor clearance	[115]
*MST1P2*	Cisplatin-resistant BC cell lines	↑	*miR-133b*/SIRT1/p53 signaling axis	Mediates cisplatin resistance in experimental models; no clinical predictive validation	[72]
*LINC00857*	TCGA and MiTranscriptome datasets; BC cell lines	↑	LMAN1-associated regulation	Associated platinum non-response in small retrospective datasets; biomarker-by-treatment interaction not established	[116]
*MALAT1*	BC tissues; BC cell lines	↑	*miR-101-3p*/VEGF-C signaling axis	Promotes cisplatin resistance in experimental models; clinical predictive utility not established	[61,118]
*NEAT1*	Cisplatin-sensitive/resistant BC cells	↑	Wnt/β-catenin signaling pathway	Contributes to chemoresistance in experimental models	[119]
*TUG1*	BC tissues; irradiated cells; xenograft model	↑	HMGB1-mediated regulation	Promotes radioresistance in preclinical models; predictive clinical utility not established	[122]
*MAFG-AS1*	EVs; BC tissues; pembrolizumab-treated patients	↑	Immune/tumor microenvironment-associated regulation	Associated with pembrolizumab response in an exploratory treatment-annotated study; independent validation required	[130]
*FGFR-AS1*	BC tissues; BC cell lines	↑	FGFR3-associated regulation	Promotes proliferation, migration, and tumor progression; potential pathway-context marker rather than a validated treatment selector	[73]

Abbreviations: BC, bladder cancer; BCG, bacillus Calmette–Guérin; ceRNA, competing endogenous RNA; EVs, extracellular vesicles; EZH2, enhancer of zeste homolog 2; FGFR3, fibroblast growth factor receptor 3; HMGB1, high mobility group box 1; LMAN1, lectin mannose binding 1; lncRNA, long non-coding RNA; MIBC, muscle-invasive bladder cancer; miRNA (miR), microRNA; NMIBC, non-muscle-invasive bladder cancer; PRC2, polycomb repressive complex 2; SIRT1, sirtuin 1; TCGA, The Cancer Genome Atlas; TERC, telomerase RNA component; TERT, telomerase reverse transcriptase; ↑, upregulation.

## Data Availability

No new data were created or analyzed in this study. Data sharing is not applicable to this article.

## Abbreviations

The following abbreviations are used in this manuscript:

AUC	area under the receiver-operating characteristic curve
AUA	American Urological Association
BC	bladder cancer
BCG	bacillus Calmette–Guérin
BCYRN1	brain cytoplasmic RNA 1
CCDC148-AS1	CCDC148 antisense RNA 1
ceRNA	competing endogenous RNA
CIS	carcinoma in situ
CT	computed tomography
CTCs	circulating tumor cells
DDP	cisplatin
EAU	European Association of Urology
EMT	epithelial–mesenchymal transition
EVs	extracellular vesicles
EZH2	enhancer of zeste homolog 2
FFPE	formalin-fixed paraffin-embedded
FGFR	fibroblast growth factor receptor
FGFR3-AS1	FGFR3 antisense RNA 1
GAS5	growth arrest-specific transcript 5
H3K27	histone H3 lysine 27
H3K27me3	histone H3 lysine 27 trimethylation
HK2	hexokinase 2
HMGB1	high mobility group box 1
HOTAIR	HOX transcript antisense RNA
HR	hazard ratio
HYMA1	HYMAI imprinted transcript
IFNγ	interferon-gamma
INCR1	interferon-stimulated noncoding RNA 1
LINC00857	long intergenic non-protein coding RNA 857
LMAN1	lectin mannose-binding 1
lncRNA	long non-coding RNA
MAFG-AS1	MAFG antisense RNA 1
MALAT1	metastasis-associated lung adenocarcinoma transcript 1
MIBC	muscle-invasive bladder cancer
MIR4435-2HG	MIR4435-2 host gene
miR, miRNA	microRNA
mRNA	messenger RNA
MST1P2	macrophage-stimulating 1 pseudogene 2
NAC/AC	neoadjuvant chemotherapy/adjuvant chemotherapy
ncRNA	non-coding RNA
NEAT1	nuclear paraspeckle assembly transcript 1
NGS	next-generation sequencing
NMIBC	non-muscle-invasive bladder cancer
NMP22	nuclear matrix protein 22
NPV	negative predictive value
PCAT-1	prostate cancer-associated transcript 1
PCR	polymerase chain reaction
PD-1	programmed cell death protein 1
PD-L1	programmed death-ligand 1
PRC2	polycomb repressive complex 2
PVT1	plasmacytoma variant translocation 1
qRT-PCR	quantitative reverse-transcription polymerase chain reaction
RFS	recurrence-free survival
RMRP	RNA component of mitochondrial RNA-processing endoribonuclease
RNA	ribonucleic acid
ROC	receiver-operating characteristic
RT-RAA	reverse transcription recombinase-aided amplification
SCARNA10	small Cajal body-specific RNA 10
SIRT1	sirtuin 1
SNHG16	small nucleolar RNA host gene 16
SPRY4-IT1	SPRY4 intronic transcript 1
STAT3	signal transducer and activator of transcription 3
SUMO1P3	small ubiquitin-like modifier 1 pseudogene 3
SUZ12	suppressor of zeste 12
SVM	support vector machine
TCGA	The Cancer Genome Atlas
TERC	telomerase RNA component
TERT	telomerase reverse transcriptase
TIDE	tumor immune dysfunction and exclusion
TUG1	taurine-upregulated gene 1
TUR	transurethral resection
TURBT	transurethral resection of bladder tumor
UBC1	ubiquitin C pseudogene 1
UCA1	urothelial carcinoma-associated 1
uEVs	urinary extracellular vesicles
UNMIBC	up-regulated in non-muscle-invasive bladder cancer
UTI	urinary tract infection
VEGF-C	vascular endothelial growth factor C
Wnt	wingless/integrated signaling pathway
